# Content validity of the electronic faces thermometer scale for pain in children: is a picture worth more than a thousand words?

**DOI:** 10.3389/fpain.2024.1372167

**Published:** 2024-04-11

**Authors:** Angelica Höök, Charlotte Castor, Maria Björk, Emma Forsgren, Anders Muszta, Stefan Nilsson

**Affiliations:** ^1^ Institute of Health and Care Sciences, Sahlgrenska Academy, University of Gothenburg, Gothenburg, Sweden; ^2^ University of Gothenburg Centre for Person-Centred Care (GPCC), Sahlgrenska Academy, University of Gothenburg, Gothenburg, Sweden; ^3^ Department of Health Sciences, Faculty of Medicine, Lund University, Lund, Sweden; ^4^ The CHILD Research Group, Department of Nursing, School of Health and Welfare, Jönköping University, Jönköping, Sweden; ^5^ Institute of Medicine, Sahlgrenska Academy, University of Gothenburg, Gothenburg, Sweden; ^6^ School of Public Health and Community Medicine, University of Gothenburg, Gothenburg, Sweden; ^7^ Queen Silvia Childreńs Hospital, Sahlgrenska University Hospital, Gothenburg, Sweden

**Keywords:** e-health, children, pain assessment, hypothetical pain, think-aloud, person-centered care

## Abstract

**Introduction:**

Early recognition of pain in children is crucial, and their self-report is the primary source of information. However, communication about pain in healthcare settings can be challenging. For non-verbal communication regarding different symptoms, children prefer digital tools. The electronic Faces Thermometer Scale (eFTS) utilizes a universal design with colors, face emojis, and numbers on an 11-point scale (0–10) for pain assessment. The aim of this study was to establish content validity of the eFTS for pain assessments in children.

**Methods:**

A mixed methods design was used. The study took place at a university hospital in eastern Sweden, involving 102 children aged 8–17 years who visited outpatient clinics. Participants were presented with 17 pictures representing varying pain levels and asked to assess hypothetical pain using the eFTS. A think-aloud approach was employed, prompting children to verbalize their thoughts about assessments and the eFTS. Quantitative data were analyzed using descriptive and comparative statistics, together with a qualitative approach for analysis of think-aloud conversations.

**Results:**

A total of 1,734 assessments of hypothetical pain using the eFTS were conducted. The eFTS differentiated between no pain (level 0–1) and pain (level 2–10). However, no clear agreement was found in the differentiation between hypothetical pain intensity levels (level 2–10). The analysis revealed that children utilized the entire scale, ranging from no pain to high pain, incorporating numbers, colors, and face emojis in their assessments.

**Discussion:**

The variability in assessments was influenced by prior experiences, which had an impact on the statistical outcome in our study. However, employing the think-aloud method enhances our understanding of how children utilize the scale and perceive its design, including the incorporation of emotion-laden anchors. Children express a preference for using the eFTS to assess their pain during hospital visits.

## 1 Introduction

Pain is described by the International Association for the Study of Pain as “an unpleasant sensory and emotional experience associated with, or resembling that associated with, actual or potential tissue damage” (1). During hospitalization, it is common for children to experience pain (2). Despite evidence-based guidelines, a substantial proportion of pediatric patients do not receive optimal pain treatment. Regular assessments as well as evaluation of individual response to treatment are advised in order to prevent undertreatment of pain. All children have the right to express their opinion as outlined in Article 12 of the United Nation's Convention on the Rights of the Child (3). According to the same convention, they have the right to be heard and to be taken seriously. Within the Lancet Child and Adolescent Health Commission, the stated goals are to make pain matter, understood, visible and better—meaning that management needs to improve in several ways, for example, by making sure children have access to evidence-based pain assessments (4). Despite this, in the context of a Norwegian postoperative setting, only 19% of the children were instructed to utilize a pain assessment tool for reporting their pain (5). Children do not always tell the nurses that they are in pain; they sometimes wait for the nurse to ask them or think that the nurse can recognize if they are in pain (6).

Studies have shown that pain assessments conducted by nurses elicit lower scores compared to assessments provided by parents or the children themselves (7, 8). Accordingly, challenges arise when conducting pain assessments in children. Earlier experiences among children influence their interpretation of top anchors in assessment scales (9). Individual differences in maturity should also be taken into consideration—children develop differently (10). A person-centered approach may make it easier for children to give opinions about their care. Within person-centered care, the patient is seen as a partner in healthcare with shared decision-making (11). To become a partner, there is a need for communication between patients and healthcare professionals. Communication with children in hospital can be challenging due to their age, language barriers and cognitive development. Children may lack the ability to understand healthcare professionals as well as the ability to communicate about their symptoms (12). To facilitate children's communication, pictorial support may be used to support both non-verbal and verbal communication (13). Furthermore, children prefer digital tools for non-verbal communication about different symptoms (12).

To find out if a method of measuring pain is reliable and accurate in adults, there are procedures involving an experimental approach. In this process, adults use the pain measure to evaluate the intensity of pain sensations caused by an unpleasant stimulus, like thermal pulses with different temperatures (14). To establish validity in an assessment scale for children, different procedures must be employed, and hypothetical pain can be used for validation purposes and to establish changes in pain intensity without any unpleasant stimuli (15, 16).

Using hypothetical scenarios has served as a methodology for evaluating and improving self-report scales designed to measure pain intensity. When using hypothetical pain, there is an opportunity to detect alteration in the pain assessment corresponding to changes in pain levels with the same participant. Participants are asked to assess or describe the pain they think is presented in a picture or verbal vignette and are then asked how much pain they would have if they were in that same situation (15, 17).

Digital assessments offer new opportunities to promote measurement compared to the analogue scales that have traditionally been used in healthcare. The electronic Faces Thermometer Scale (eFTS) was developed as an e-health tool available for mobile phones and tablet computers, where the user can self-report their pain, among other symptoms. The development of eFTS was inspired, among other things, by previously validated scales designed for children's use (16, 18). It uses a universal design including colors, numbers, and face emojis to differentiate levels of pain intensity and may enhance children's self-reporting of pain (13). There are ongoing studies to test the agreement between the eFTS, the Coloured Analog Scale (CAS) and the Faces Pain Scale-Revised (FPS-R) (19). The aim of the present study was to establish content validity of the eFTS for pain assessments and the research questions were:

- Examine levels of agreement between eFTS and predetermined categories of hypothetical pain intensity.
- Examine the acceptability and comprehensibility of eFTS.

## 2 Materials and methods

### 2.1 Design

**Figure 1:**
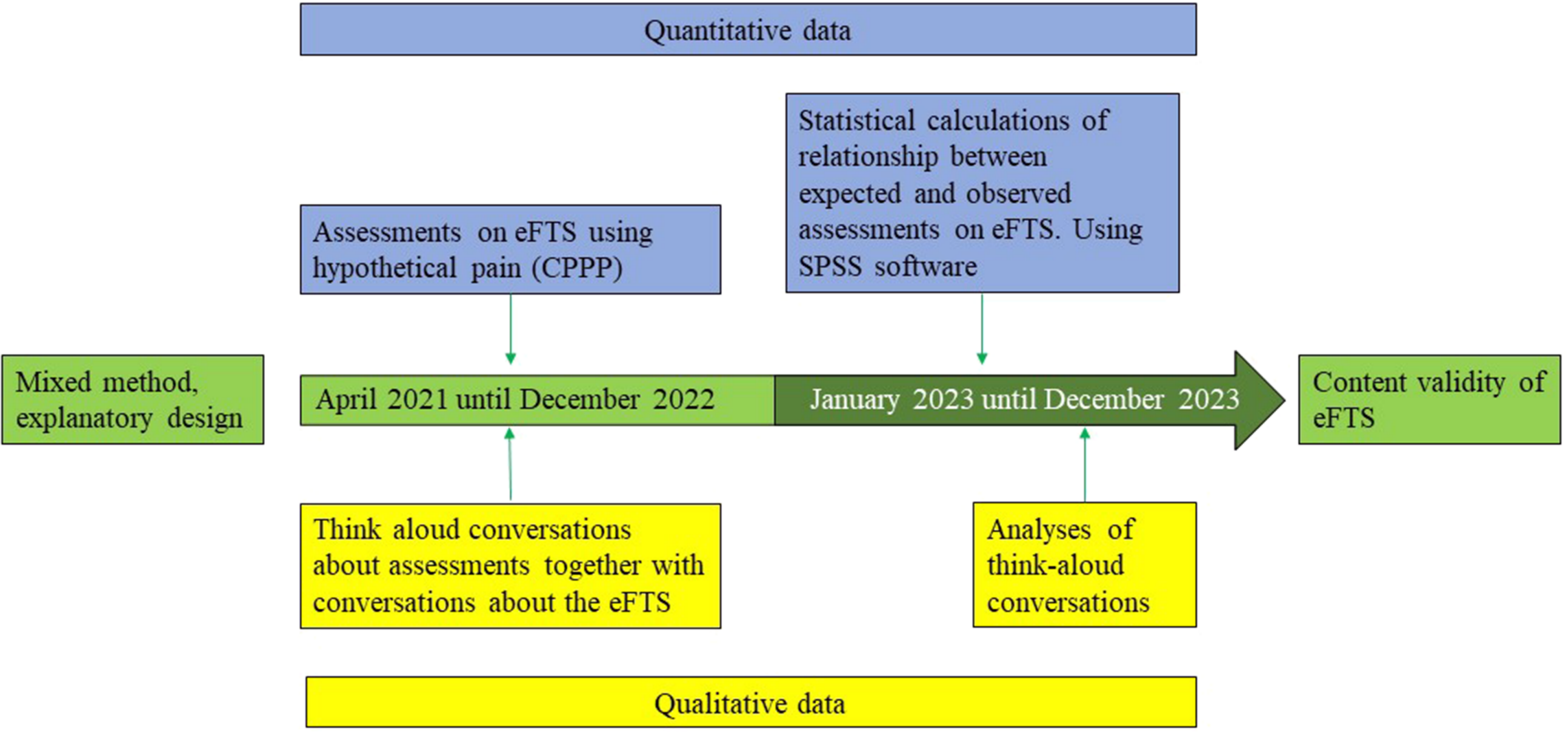
Flowchart displaying study design and data collection.

This study uses a mixed method-explanatory design. The study is a single center study and a part of the international eFTS validation project (19). Descriptive and comparative statistical analysis was used, together with a qualitative approach for analyzing think-aloud conversations. See Figure 1 for a flowchart describing the process.

### 2.2 Setting

The study was carried out in a pediatric department at a university hospital in eastern Sweden, serving children up to 18 years old. Children visiting the outpatient oncology unit (e.g., for bone marrow aspirations, lumbar punctures, or oncology treatments) or the outpatient non-oncology units (e.g., for infusions, injections, or minor surgeries) were invited to participate.

### 2.3 Instruments

#### 2.3.1 Pain assessment scale [electronic faces thermometer scale (eFTS)]

**Figure 2:**
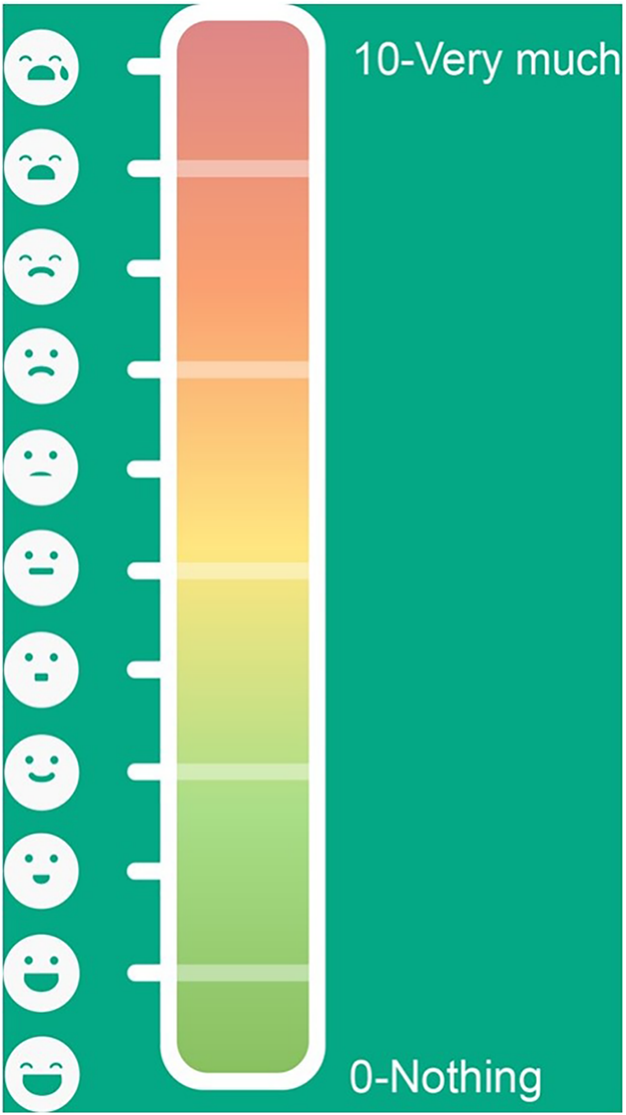
The electronic faces thermometer scale (eFTS). The eFTS uses face-emojis, colors and numbers as indicators for pain intensity levels.

The eFTS, available in a smartphone or in a tablet computer, uses colors ranging from green to red; numbers, 0 (nothing) to 10 (very much); and face emojis where green is symbolized with a happy face emoji and red is symbolized with a sad face emoji (19) (Figure 2).

#### 2.3.2 Hypothetical pain [charleston pediatric pain pictures (CPPP)]

The Charleston Pediatric Pain Pictures (CPPP) consist of 17 drawn pictures displaying a young child, non-specific regarding gender and lacking any facial expression, engaged in some activity that might be painful. When developing the CPPP, a total of 27 possible painful situations were illustrated. The final 17 pictures were selected based on ratings by six experienced child clinicians. The pictures that were selected were those where there was agreement among the raters regarding their placement on one of the four pain intensity levels (20). Table 1 shows a description of the pictures included and their predetermined order of presentation.

**Table 1:** CPPP description of pictures and displayed activities for each pain category together with their predetermined order of presentation.

Pain category	Numbers of pictures	Description of picture and order of appearance
No pain (NP)	4	(1) Looking at a picture book at home, (7) Having temperature taken by a nurse, (14) Doctor listening to heart, (17) Playing a game with friends at home
Low pain (LP)	4	(4) Having a Band-Aid removed, (8) Being pushed down by a playmate, (10) Being pinched by a playmate, (15) Tripping and falling on the carpet at home
Moderate pain (MP)	5	(3) Stubbing toe on sidewalk, (6) Hitting head on table at home, (11) Scraping knee on sidewalk, (13) Having a book fall on foot, (16) Falling down steps at home
High pain (HP)	4	(2) Burning hand on stove at home, (5) Receiving a shot at the doctor's office, (9) Being stung by a bee outside, (12) Stepping on a nail outside

The pictures were initially created for evaluating hypothetical pain in healthy children aged 3–6 years old. Subsequently, the CPPP have been validated for use in both preschool and school-aged children, spanning the age range of 5–9 years (17, 20). They have also been used for establishing psychometric properties in other ages, e.g., the Northern Pain Scale includes participants from 5 to 83 years old (21). Each picture is presented together with a vignette explaining the situation (20).

The vignettes were forward and back translated. First, the four native Swedish speaking authors (A.H., C.C., M.B. and S.N.) with English proficiency and skilled in nursing research translated the vignettes from English to Swedish and also reviewed for cultural challenges and made adaptions. The vignettes were then translated back to English by a professional translator whose native language is English but who speaks Swedish fluently. Lastly, the translated version was reviewed, and minor adjustments were made. One example is the word “mad” in the sentence “A bee got mad and stung you on the arm” was first translated to the Swedish word “arg”, then back translated to the English word “angry”, the final word which was used was the Swedish word “ilsket”.

### 2.4 Participants

A consecutive sampling strategy was used, and the sample set was set to 100 children. Inclusion criteria were children between 8 and 17 years old, who were able to read and understand Swedish and English and were visiting the hospital for a planned procedure. Exclusion criteria were children or parents who did not understand the instructions including the concept of hypothetical pain. A total of 124 children were invited from April 2021 until December 2022. Twenty-two children declined participation due to lack of time, not being in the mood or feeling too fatigued. The interviews from two participants were incomplete.

Studies validating CPPP included younger children (3–6 years, mean 4.4 and 5–10 years, mean 6.9) with a sample set between 50 and 58 children (17, 20).

The concept of “children” has been a subject of ongoing debate in recent years, with advancements in our understanding of cognitive development partially shaping the delineation of age groups. Traditionally, adolescence has been categorized as spanning from 10 to 19 years; however, a report from The Lancet suggests that adolescence may commence earlier, even as early as 8 years old. Concurrently, the term “teenage” (pertaining to ages 13–19 years) is also commonly employed (22).

In the context of this study, the primary objective was to segment the sample across various age brackets to discern potential distinctions within this critical developmental phase. Accordingly, we opted to categorize participants into four distinct age groups, i.e., 8–10 years, 11–12 years, 13–15 years and 16–17 years.

The sample size determination was informed by three prior studies within the same age bracket, all aiming to validate an eleven-grade scale through various methodologies. The initial investigation, employing the CAS which is akin to the eFTS, incorporated 104 children aged between 5 and 16 years (16). Subsequently, a comparative study of four distinct scales—namely, the plasticized color analogue scale, a paper visual analogue scale, the paper-based Wong-Baker FACES Pain Rating Scale, and a verbal numeric scale—enrolled 87 children aged 8–18 years (23). Lastly, a study involving 83 children within the same age range employed three diverse pain assessment tools: the Numeric Rating Scale, The Verbal Rating Scale, and the facial expression-based scales (specifically, the FPS-R and the Facial Affective Scale) (24). Given these precedents, we elected to include a cohort of 100 children in this study.

### 2.5 Study procedures and data collection

Children included in the study were informed of the study details within a time frame ranging from 30 min to four weeks before its commencement and they were asked to participate during their pre-planned stay at the outpatient clinic. After receiving written informed consent and assent from the children and their parents, data collection procedure began. Data collection was performed during an infusion, before or after a procedure. All data collection was conducted by the first author, AH, who has two decades of experience in pediatric healthcare and pain management and is concurrently pursuing a PhD. Participating children were situated either in their hospital bed or in a room at the outpatient unit during the data collection. One or two parents accompanied all participants who were between 8 and 14 years old, while some —but not all— of the 15–17-year-old participants were accompanied by their parents. Visits at the outpatient clinic lasted for between one and eight hours. The data collection lasted from 8 to 25 min, with a mean of 13 min.

Participants were introduced to the data collection procedure by AH reading a prelude explaining eFTS and CPPP in Swedish.

“Participation in this study means that you will get to look at 17 pictures. Each picture shows different everyday situations with different degrees of pain. When you look at the picture, I would like you to imagine that it’s you in the picture, and then I’m going to read a short text about each picture, and then you’re going to mark on this assessment scale on the tablet computer how much it hurts. At the top there’s a sad face and it’s red and it says ten; it represents a lot of pain. At the bottom is a happy face; it says zero and it is green, which represents no pain. You click on the face that best matches your emotion for that picture.”

Participating children were instructed to examine the pictures, one at a time, in a predetermined order. AH read the explanatory vignette assigned to the particular picture and they were instructed to assess their hypothetical pain. Data was collected stepwise:

(1) The participant assessed their hypothetical pain in the eFTS accessible on a tablet computer.
(2) The participant described verbally how they reasoned when using the eFTS to assess their hypothetical pain and the conversations were audio recorded.
(3) Participants were asked “would you like to use this scale to assess your pain when you are in pain yourself?” after having finished their assessment of hypothetical pain.

The utilization of the think-aloud method aims to illuminate the cognitive processes occurring in an individual’s mind while engaging in a task. The participants are prompted to vocalize their thoughts while solving a problem and are encouraged to verbalize their spontaneous thoughts (25, 26). Demographic data on age and gender information was collected through a questionnaire in the beginning of data collection.

### 2.6 Analyses

To characterize participants and assess whether eFTS could distinguish between levels of pain intensity, descriptive and comparative statistics were employed. The participants' verbal descriptions of their assessments were transcribed and analyzed.

#### 2.6.1 Quantitative analyses

**Figure 3:**
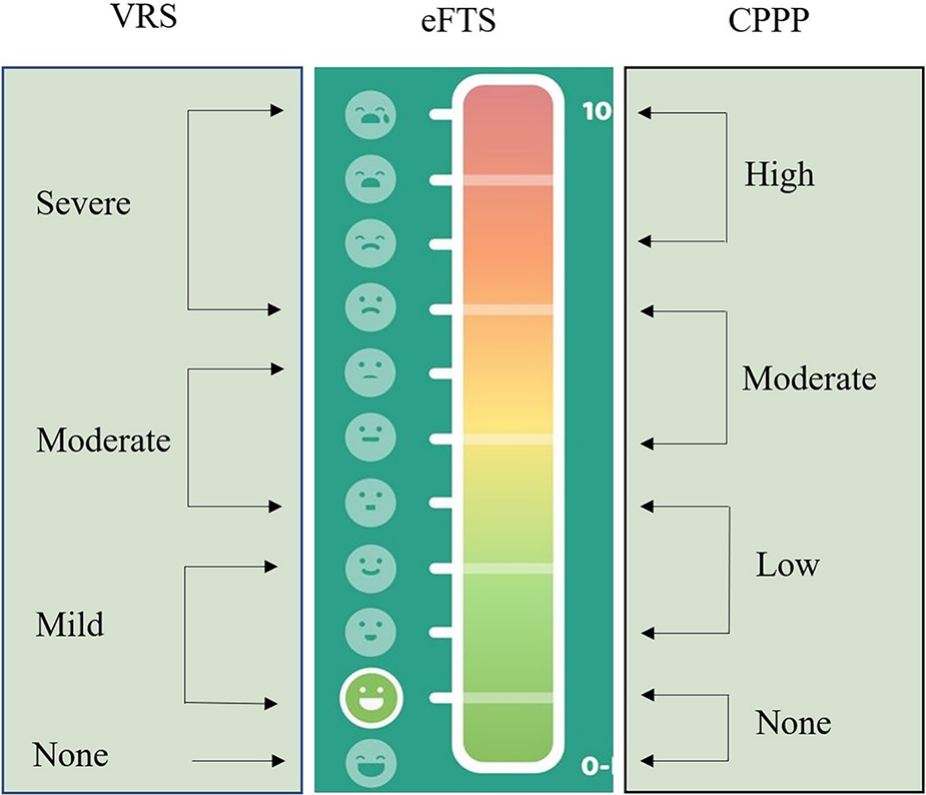
Values on eFTS comparing values retrieved from VRS 4 and values of pain intensity levels on CPPP.

The agreement between observed values, i.e., children's assessments on eFTS, and expected values in CPPP were analyzed with a chi-square test based on numerical scores. Numerical scores for observed values were retrieved via assessments on eFTS. To transform hypothetical pain intensity levels used in CPPP into numerical scores the Verbal Rating Scale (VRS) 4 was used with an adjustment for school aged children (Figure 3) (17). The VRS is a scale using verbal descriptors for pain intensity. There are different versions according to the number of verbal descriptors and VRS 4 has been used for retrieving expected values, with pain intensity described as none, mild, moderate, and severe (27).

In our analysis we used a modification of the standard chi-square test. Our test statistic is the median chi-square distance of the individual comparisons between eFTS and the numerical values for CPPP, instead of the usual sum of all individual comparisons. The rationale for using the median instead of the sum is that the median chi-square distance better represents the performance of the majority of the children; if a few children perform very differently than expected, this has a major impact on the sum, whereas the median is unaffected. If the test statistic falls within a 95% reference interval of the chi-square distribution with one degree of freedom, i.e., the interval (0–1.03), then this indicates that the children's assessment on eFTS corresponds with hypothetical pain intensity levels in CPPP. Analyses were completed using The Statistical Package for Social Sciences (IBM SPSS Statistics 29).

#### 2.6.2 Qualitative analyses

Think-aloud conversations were organized according to the pictures they were related to or according to the question related to thoughts about using the scale. Conversations associated with each picture were analyzed together and conversations associated with comprehensibility and acceptability of the eFTS were analyzed separately and further divided into conversations about the use of the scale and conversations about the design of the scale.

The analyses were descriptive with a low level of interpretation of the text (26). The texts corresponding to each picture were thoroughly and repeatedly read to identify commonalities in how the children reasoned about their assessments. The conversations were summarized, and specific words or meanings associated to assessments or to the eFTS that were used repeatedly by different participants were identified.

#### 2.6.3 Ethical considerations

The study was approved by the regional ethical review board (Dnr 2020-051119, 2021-01213). Informed written consent was obtained from the accompanying parent for the children between 8 and 14 years old and directly from participating children who were between 15 and 17 years old. In cases where there were two caregivers but only one accompanying the child, informed written consent was obtained from the non-accompanying parent through e-mail or SMS. Written assent was obtained from participating children aged between 8 and 14 years old.

## 3 Results

### 3.1 Demographic data

The study is based on 58 boys and 44 girls with an average age of 12.5 years with a standard deviation of 2.7 years. To reach an understanding of differences in assessments according to age, the data set was divided into four balanced age groups based on quartiles of age distribution (Table 2).

**Table 2:** Distribution of participants.

Category	*N*	(%)	Mean age (SD)
All participants	102	(100)	12.5 (2.7)
Age group			
8–10	28	(27)	9.2 (0.9)
11–12	24	(24)	11.5 (0.5)
13–15	33	(32)	14.0 (0.8)
16–17	17	(17)	16.4 (0.5)

### 3.2 Agreement between assessments using the eFTS and hypothetical pain intensity

A total of 1,734 assessments on the eFTS from participating children were obtained (i.e., 102 children assessing 17 pictures each). In pictures displaying no pain, an agreement was seen between assessments using eFTS and expected assessments. For pictures displaying hypothetical pain at other pain levels (low, moderate, and high pain), a variation in agreements between eFTS assessments and expected assessments was seen. Variations according to the different pain levels are shown in Table 3.

**Table 3:** Md chi-square distances for each pain level displayed in CPPP.

Hypothetical pain intensity level	Number of pictures	Md chi-square distances
No pain	4	1.02
Low pain	4	11.63
Moderate pain	5	8.71
High pain	4	3.57

The psychometric analysis of each of the pictures representing different pain intensity levels are displayed in Tables 4–7. If observed values agrees with expected values, there is an agreement between assessments on eFTS and pain intensity levels in CPPP.

**Table 4:** No pain pictures, median chi-square distances (Md chi²) together with number of assessments (*N*) at pain level 0–1 on the eFTS.

	Picture 1 Looking at a picture book at home			Picture 7 Having temperature taking by a nurse			Picture 14 Doctor listening to heart			Picture 17 Playing a game with friends			Overall agreement, no pain pictures
Age group	Md chi²	N/total	(%)	Md chi²	N/total	(%)	Md chi²	N/total	(%)	Md chi²	N/total	(%)	Md of md chi²
8–10	**0** **.** **28**	21/28	(75)	**0**.**28**	20/28	(71)	**0**.**28**	24/28	(86)	**0**.**28**	26/28	(93)	**0**.**28**
11–12	**0**.**24**	20/24	(83)	**0**.**24**	20/24	(83)	**0**.**24**	22/24	(92)	**0**.**24**	23/24	(96)	**0**.**24**
13–15	**0**.**33**	28/33	(85)	**0**.**33**	32/33	(97)	**0**.**33**	32/33	(97)	**0**.**33**	29/33	(88)	**0**.**33**
16–17	**0**.**17**	16/17	(94)	**0**.**17**	17/17	(100)	**0**.**17**	17/17	(100)	**0**.**17**	16/17	(94)	**0**.**17**

Bold numbers indicate distances between 0 and 1.03.

**Table 5:** Low pain pictures, median chi-square distances (Md chi²) together with number of assessments (*N*) at pain level 2–4 on the eFTS.

	Picture 4 Having a Band-Aid removed			Picture 8 Being pushed down by a playmate			Picture 10 Being pinched by playmate			Picture 15 Tripping and falling on a carpet at home			Overall agreement, low pain pictures
Age group	Md chi²	N/total	(%)	Md chi²	N/total	(%)	Md chi²	N/total	(%)	Md chi²	N/total	(%)	Md of md chi²
8–10	2.52	13/28	(46)	5.14	9/28	(32)	5.14	8/28	(29)	2.52	11/28	(39)	3.83
11–12	2.41	12/24	(50)	6.45	9/24	(38)	2.65	12/24	(50)	2.41	16/24	(67)	2.53
13–15	6.62	14/33	(42)	3.79	14/33	(42)	3.79	12/33	(36)	5.11	18/33	(55)	4.45
16–17	3.46	8/17	(47)	2.03	7/17	(41)	**0** **.** **31**	8/17	(47)	4.05	10/17	(59)	2.74

Bold numbers indicate distances between 0 and 1.03.

**Table 6:** Moderate pain pictures, median chi-square distances (Md chi²) together with number of assessments (*N*) at pain level 5–7 on the eFTS.

	Picture 3 Stubbing toe on sidewalk			Picture 6 Hitting head on table at home			Picture 11 Scraping knee on sidewalk			Picture 13 Having a book fall on foot			Picture 16 Falling down steps at home			Overall agreement, moderate pain pictures
Age group	Md chi²	N/total	(%)	Md chi²	N/total	(%)	Md chi²	N/total	(%)	Md chi²	N/total	(%)	Md chi²	N/Total	(%)	Md of md chi²
8–10	**0** **.** **83**	15/28	(54)	1.85	15/28	(54)	1.85	14/28	(50)	1.85	14/28	(50)	1.85	15/28	(54)	1.85
11–12	3.98	7/24	(29)	**0**.**80**	17/24	(71)	2.41	13/24	(54)	2.41	12/24	(50)	2.65	8/24	(33)	2.41
13–15	2.67	15/33	(45)	1.36	16/33	(48)	3.80	16/33	(48)	3.80	16/33	(48)	1.36	18/33	(55)	2.67
16–17	2.03	7/17	(41)	**0**.**31**	10/17	(59)	3.46	9/17	(53)	**0**.**17**	8/17	(47)	4.05	8/17	(47)	2.03

Bold numbers indicate distances between 0 and 1.03.

**Table 7:** High pain pictures, median chi-square distances (Md chi²) together with number of assessments (*N*) at pain level 8–10 on the eFTS.

	Picture 2 Burning hand at a stove at home			Picture 5 Receiving a shot at the doctor's office			Picture 9 Being stung by a bee outside			Picture 12 Stepping on a nail outside			Overall agreement, high pain pictures
Age group	Md chi²	N/total	(%)	Md chi²	N/total	(%)	Md chi²	N/total	(%)	Md chi²	N/total	(%)	Md of md chi²
8–10	**0** **.** **28**	23/28	(82)	10.56	10/28	(36)	1.56	14/28	(50)	**0**.**28**	25/28	(89)	**0**.**92**
11–12	**0**.**80**	14/24	(58)	12.91	3/24	(13)	3.98	9/24	(38)	**0**.**24**	22/24	(92)	2.11
13–15	1.75	19/33	(58)	20.41	4/33	(12)	8.45	10/33	(30)	**0**.**33**	30/33	(91)	4.39
16–17	2.64	11/17	(65)	5.27	1/17	(6)	5.27	2/17	(12)	**0**.**17**	14/17	(82)	3.96

Bold numbers indicate distances between 0 and 1.03.

#### 3.2.1 No pain pictures

The four pictures displaying no pain all worked as intended. Observed assessments were all within a 95% reference interval in each age category with median chi-square distances between 0 and 1.03 (Table 4).

Think-aloud conversations were about the absence of pain in the pictures displayed. Nevertheless, some of the participants mentioned discomfort when sitting on the floor reading a book:

“Nothing, because, you know, you hardly move; unless you have some kind of injury before, it doesn't hurt.” (picture 1, assessed 0 on eFTS) 11-year-old.

“If I'm reading, and I'm sitting, well, after a little while, it would start to hurt.” (picture 1, assessed 2 on eFTS) 16-year-old.

“There, you don't really have much pain, right?” (picture 17, assessed 1 on eFTS) 9-year-old.

#### 3.2.2 Low pain pictures

All four pictures displaying low pain had assessments outside the reference interval with differences in age groups. In the oldest age group (16–17 years), there was an agreement between eFTS and CPPP in picture number 10. In the younger age groups (8–10 years, 11–12 years and 13–15 years), there were weaker agreements in this pain intensity level. Assessments regarding picture number 8, displaying a child being pushed, had less than 40% assessments within intended interval 2–4 on the eFTS (Table 5).

Certain circumstances were mentioned that had an impact on the assessment process. When assessing this picture (number 8), children reasoned about falling in terms of how hard the ground was and in what way you hit the ground. Previous experiences from similar situations were mentioned in the conversations:

“Yes, but then I ran into a rock.” (picture 8, assessed 5 on eFTS) 8-year-old.

“It also depends, sometimes it's fun, and sometimes…well, it hurts.” (picture 8, assessed 5 on eFTS) 12-year-old.

“But it would probably hurt the head, falling down like that” (picture 15, assessed 4 on eFTS) 11-year-old.

#### 3.2.3 Moderate pain pictures

Moderate pain was displayed in five pictures, none of which were within the 95% reference interval in the age groups, meaning a median chi-square distance between 0 and 1.03 (Table 6). The number of assessments within the intended interval (5–7 on eFTS) was low, indicating a variety in assessments. For example, in the age group 11–12 years there were variations in assessments in picture number 3 and picture number 16 with few observed assessments within the intended interval.

Think-aloud conversations also showed variety in terms of how participants thought about the situations. Some of the participants talked about pain intensity and/or how long it would hurt. Consequences of the fall in picture number 16 were also mentioned and explained participants’ assessments.

“Maybe, I wouldn't say it hurts a lot, but specifically the toes, it does hurt quite a bit, but it's quite brief, so…” (picture 3, assessed 8 on eFTS) 11-year-old.

“Yes, it's really the worst pain, to the point where you could cry about it.” (picture 11, assessed 9 on eFTS) 16-year-old.

“Alternatively, you could break something; if you break something, it's a ten out of ten. But let's say you don't break anything, you just hit it, and that can still be really painful.” (picture 16 assessed 8 on eFTS) 10-year-old.

“But I probably wouldn't say that it hurts all that much.” (picture 16, assessed 5 on eFTS) 15-year-old.

#### 3.2.4 High pain pictures

The assessments for this pain intensity level varied; for picture number 12, there was agreement between observed and expected assessments. Regarding picture number 2, more than 50% of the assessments were within the intended interval — and in the younger age groups, there was agreement between observed and expected assessments. Observed assessments on picture number 5 and picture number 9 were outside the reference interval in all age groups with high median chi-square distances (Table 7). A total of 84 (82%) participants assessed this picture as representing lower pain than expected.

Conversations regarding picture number 5 included reasoning about different strategies used during injections and previous experiences such as the use of numbing cream:

“It depends on if I had anesthetic cream.” (picture 5, assessed 4 on eFTS) 9-year-old.

“But I have got used to having injections because I have had a lot lately, I would consider it easy to have an injection because it does not hurt for long.” (picture 5, assessed 4 on eFTS) 16-year-old.

Regarding picture number 9, the duration of pain in combination with the lack of experience were discussed:

“Yeah, about a five then. I haven't been stung very often, but I have been stung, and it wasn't pleasant…it's like…it stings quite a bit, but it's not something that really hurts, and it goes away after a while.” (picture 9, assessed 5 on eFTS) 16-year-old.

“No, I've never been stung, but I guess I'll try to imagine this. It's maybe, um, right there.” (picture 9, assessed 6 on eFTS) 14-year-old.

### 3.3 Acceptability and comprehensibility of eFTS

Of the 102 participating children, 100 stated that the eFTS would work as an assessment scale which they would like to use if they were in pain. The other two participants thought that they did not want to use any scale at all when talking about pain. The eFTS was displayed on a tablet computer and all participating children understood how eFTS worked and how they should use it when assessing hypothetical pain.

#### 3.3.1 Overall usage

The children discussed whether eFTS could facilitate communication about pain. They thought that the eFTS was easy to use and addressed the fact that if they did not want to tell the healthcare professionals, they could just press on the tablet computer:

“So, I think it is very, very good to use that thermometer at the hospital.” 11-year-old.

“If I don't want to tell, I can just press there.” 8-year-old.

“Well, yes, or it kind of shows how much pain you're in, so more people might understand then.” 17-year-old.

#### 3.3.2 Universal design

Some children preferred to use numbers or colors, whereas others preferred face emojis to assess their hypothetical pain. The children thought that the faces were similar and discussed the usefulness of the colors, where green was good and red was bad. Choosing numbers between zero and ten could be helpful. The face emojis were used to show feelings. Some of the children also used a combination of the three to express their pain or discomfort. They had personal preferences based on how they most easily expressed their pain intensity. Each child chose their preference for assessing the pictures:

“Because I thought the faces were quite similar on…well, not very, but I thought it felt easier for…yeah, because I think green is good and red is bad, you know. The faces were kind of in between, sort of.” 15-year-old.

“That is very good because you can also see…you can kind of imagine, like, between zero and ten, but you can also see here on…I mean, the faces, roughly how it feels.” 14-year-old.

 “It was easier to count. Because, well, I know from zero to ten.” 11-year-old.

## 4 Discussion

The aim of this study was to establish content validity of the eFTS for pain assessments in children. Quantitative analyses showed a clear agreement between eFTS and CPPP when it comes to differentiating between no pain (assessments between 0 and 1 on eFTS) and pain (assessments between 2 and 10 on eFTS) in all age groups. No clear agreement was found in the differentiation between hypothetical pain intensity levels (assessments between 2 and 10 on eFTS).

A diversity in assessments in different pictures and among different ages was seen with agreements within certain age groups and specific pictures.

Qualitative findings deepened the understanding of the children's use of various parts of the scale in terms of faces, color and numbers and also revealed how the children had found the use of the eFTS easy and desirable to facilitate pain assessments and communicate about pain.

The participating children's previous experiences had a clinically relevant impact on assessments, as evidenced when analyzing think-aloud conversations in relation to the assessments that were conducted. Reported pain intensity in the same hypothetical situations varied among the children. A personal experience of the specific painful situation can make it easier for them to assess hypothetical pain. Previous research has shown a diversity in perceptions of different painful situations (28). For younger children, it is easier to remember recent experiences. Additionally, fear might influence the child's experience of pain. If participating children relate the painful situation to fear, some will assess the pain as higher (15, 29). The findings underscore the subjective nature of pain assessments in children, influenced by individual experiences, age and maturity, as well as emotional factors such as fear. Using hypothetical pain displaying different pain intensity levels as a part of the validation process provided an opportunity to measure pain intensity without an experience of pain or an experimental approach.

Some children preferred to use numbers or colors, whereas others preferred face emojis in their assessments. The children thought that the faces were similar and discussed the usefulness of the colors, where green was good and red was bad. Choosing numbers between zero and 10 could be helpful. The face emojis could be used to show feelings as well as report pain intensity. Some of the children also used a combination of the three to express their pain. They had a personal preference based on how they most easily expressed their pain intensity. Having more than one indicator for pain intensity may have an effect on assessments. However, when assessing pictures displaying no pain, there was a strong agreement between assessments which may indicate that regardless of the number of indicators simultaneously used, children were able to effectively assess their lack of pain. In eFTS, no pain is represented with a smiling face emoji and high pain is represented by a face emoji with tears. The utilization of facial expressions in pain assessment scales has been discussed by Chambers and Craig (30). Various faces pain scales, such as the Wong-Baker Faces Pain Scale, depict smiling faces and tears, while others—like the FPS-R—feature expressions without these emotional cues (30, 31). Other scales have chosen to incorporate more personalized face emojis, known as memojis (32). Scales with smiling anchors may introduce ambiguity between affective states and pain intensity as children may rate a negative emotion as more painful. Nevertheless the Wong Baker Faces Pain Scale has demonstrated efficacy in measuring pain intensity rather than fear in children aged 7–12 (33). The use of memojis in pain assessment scales was preferred in the study by Saikiran et al. (32) in which 81.6% preferred to use the Memojis Pain Scale over the Wong-Baker Faces Pain Scale and FPS-R. Memojis used in the study by Saikiran et al. are personalized according to gender, while the face emojis used in eFTS are generic in terms of gender. Psychometric findings—along with think aloud conversations—provide support for the assertion that children aged 8 years old and above are able to measure pain intensity despite the emotionally charged anchors and without a personalization based on gender. Think aloud conversations did not reveal any difficulties regarding either the use of more than one indicator for pain intensity or the use of emotion-laden anchors.

The limited development of abstract thinking in younger children poses challenges when using hypothetical pain situations. Children younger than 10 years old have not yet developed their abstract thinking, while children older than 15 are able to think abstractly (34). Young children may encounter challenges due to verbalization skills, the nature of their thought processes or the challenge of concentrating on a problem-solving task (25). In addition to statistical calculations, the use of think-aloud conversations can be helpful and—during the validation process—may illuminate the cognitive process involved while using the eFTS.

A validated self-report scale, employed as an e-health tool easily available for children, may promote a more person-centered care where the children have control over their assessments. To facilitate person-centered care, there is a need to establish a partnership where the child can become a partner in decision-making regarding treatment and care. The patient's story-telling acts as the initial step in establishing a partnership. Person-centered care is considered to be a gold standard in healthcare and focuses on the child's best interests (12). The universal design used in eFTS has been developed together with children, parents, and healthcare professionals as part of an e-health tool to assess different symptoms (13). As a digital and validated self-report scale, the eFTS has potential to give children the autonomy to decide when and how they assess their pain and promote communication about pain and pain treatment (35).

The regular use of smartphones for communication in society as a whole, where expressive symbols like emojis are prevalent, highlights the evolving landscape of communication tools and the use of generic face emojis in eFTS may simplify pain assessments (32). The graduation in numbers and colors makes an assessment scale more easily understood with different positions reflecting different pain intensity values (16).

### 4.1 Limitations

The CPPP, developed in 1988, uses drawn pictures displaying a young child without any facial expression in different settings in different painful situations. This may make it difficult to identify with the situations displayed. Some of the pictures may be considered unsuitable for life in the 21st century, e.g., the image depicting a child experiencing a burn on the hand from direct contact with a heated stove. In contemporary settings, induction technology in stoves has become more common, decreasing the risk of excessive heat exposure. This may make it difficult to identify with the situation. This is one example of several situations that children of today would be unfamiliar with.

Challenges associated with hypothetical pain include interpretations of the scale and perceptions of the accompanying images. Think-aloud conversations revealed that some children found it difficult to identify with the child displayed in the picture and to relate to the various depicted situations.

Previous studies using CPPP have been performed in healthy preschool or school children, whereas the present study was performed with children who had a planned visit to the hospital. The participating children had had previous visits in a pediatric ward and were used to being in a hospital setting, which could have influenced their assessments.

All participants in the present study had some kind of health problem since they were patients at the hospital at the time of the data collection. Data regarding the child's former experiences of health care, hospitalizations or pain were not collected—data which may have an influence on assessments. In children, there is also a need to address chronic pain, as well as acute pain. The eFTS is not tested for assessments of chronic pain.

Parents or other carers can influence children's assessments. In this study, the data collection did not include the presence of parents or other carers—or their gender.

Collection of data was performed by the first author and can be seen as a limitation prone to bias.

## 5 Conclusion

The eFTS has potential to be used in pain assessments for children, aiming to obtain validated measurements of the child's pain experience. To establish content validity, various methodologies have been used and the functionality of the eFTS in measuring pain has been demonstrated. Statistical calculations in conjunction with interviews have provided insights into children's usage of the eFTS. The universal design with numbers, colors and face emojis has contributed to facilitating the assessment of hypothetical pain intensity. Additionally, the eFTS may also provide a way to facilitate communication about pain.

## Data Availability

The datasets presented in this article are not readily available since the General Data Protection Regulation (GDPR) applies to the data collected in this study, i.e., any information that refers to an identified or identifiable natural person. The GDPR applies in principle to every kind of operation and activity and regardless of who carries out the processing of this personal data. It thus applies to companies, associations, organizations, authorities, and private individuals. Requests to access the datasets should be directed to angelica.hook@gu.se.
